# Psychosocial factors associated with health behaviors in pregnant women of advanced maternal age in Korea

**DOI:** 10.3389/fpubh.2023.1179416

**Published:** 2023-06-15

**Authors:** Songi Jeon, Wonjung Noh

**Affiliations:** ^1^Department of Nursing, Catholic Kwandong University, Gangneung-si, Gangwon-do, Republic of Korea; ^2^College of Nursing, Gachon University, Incheon, Republic of Korea

**Keywords:** advanced maternal age, health-impairing behavior, health-promoting behavior, pregnant women, psychosocial factors

## Abstract

**Objectives:**

To determine the association between psychosocial factors and health-promoting and health-impairing behaviors in pregnant women of advanced maternal age (AMA) in Korea.

**Design:**

A cross-sectional survey study.

**Setting:**

Online survey.

**Samples:**

A total of 217 pregnant women aged 35 and older agreed to participate in the study, with 207 participants completing the self-report questionnaires.

**Methods:**

We collected self-reported data on demographic, obstetric, and psychosocial factors and prenatal health behaviors using standardized measures. We conducted a descriptive analysis of the collected data and a linear regression to identify significant associations with health-promoting and health-impairing behaviors.

**Results:**

We found that maternal–fetal attachment (β = 0.43, *p* < 0.001) and “social atmosphere” of pregnancy stress (β = 0.13, *p* = 0.047) were positively associated with prenatal health-promoting behaviors. We found that artificial conception (β =-0.16, *p* = 0.011) was negatively associated with prenatal health-impairing behaviors and that multiparity (β = 0.23, *p* = 0.001) and “maternal role” of pregnancy stress (β = 0.27, *p* = 0.003) positively associated with prenatal health-impairing behaviors.

**Conclusion:**

Health-impairing behaviors of pregnant AMA women need assessment and the importance of health-promoting behaviors for maternal and infant health need reinforcing. We recommend pregnancy stress assessments at prenatal checkups and stress relief interventions that consider cultural differences and contexts rather than standardized interventions.

## 1. Introduction

Women are delaying childbirth in advanced countries (1). In Korea, the average age of women giving birth was 32.6 years, the highest among Organization for Economic Cooperation and Development (OECD) countries in 2017 (2). Korean women aged 35 and older accounted for approximately 33.8% of all births in 2020 (3).

Poor pregnancy and childbirth outcomes, including gestational diabetes mellitus (GDM), gestational hypertensive disorders (GHDs), placenta previa, congenital malformations, miscarriage, stillbirth, premature birth, low birth weight, and postpartum hemorrhage, are common among pregnant women of advanced maternal age (AMA) (4–6). The rate of premature births and the incidence of low birth weights are on the rise in Korea (3). The increased proportion of pregnant women of AMA, and the resulting detrimental pregnancy and childbirth outcomes not only have a negative impact on maternal and child health but also contribute to personal and national economic losses because of increased healthcare costs; thus, management and support for pregnant women of AMA are required (7). Lin et al. (8) reported that pregnant women of AMA could avoid negative childbirth outcomes through management, such as proper diet and physical activity, and many studies have shown that prenatal health behaviors lead to healthy birth outcomes (9–11).

Therefore, it is necessary to explore which characteristics relate to the health behavior of pregnant women of AMA. However, most studies have focused on pregnant women of all ages, and studies on pregnant AMA women are limited. Pregnant AMA women perceived pregnancy as more threatening than young women did (12), and they tended to engage in healthier behaviors (13). Hence, applying the findings of studies on pregnant women of all ages to pregnant women of AMA can make understanding the factors influencing prenatal health behaviors difficult. Furthermore, compared with other countries, pregnant women in Korea are under a lot of social pressure, which is natural given their role as mothers to behave for the health of their fetus (14).

Previous studies have explored which factors relate to prenatal health behaviors. A meta-analysis study on pregnant women's health behavior reported age, employment, income, education, parity, maternal–fetal attachment, stress, depression, and social support as predictive factors (15). Based on this, we divided prenatal health behavior-related factors into demographic, obstetric, and psychosocial factors. As a demographic factor, the higher the level of education and income, the healthier the behaviors tended to be (16, 17). Obstetric characteristics, such as current conception type, gestational age, parity, and experience of abortion, also relate to prenatal health behavior: Pregnant women in the third trimester than those in the second trimester, and younger pregnant women than older pregnant women engaged in less healthy behaviors (13, 18). Meanwhile, some studies have considered psychosocial factors as they could otherwise enhance these components through interventions when compared with demographic and obstetric characteristics. According to previous research, psychosocial factors influence prenatal health behaviors: Pregnant women's elevated self-esteem levels, fetal attachment, and social support lead to more healthy behaviors, whereas high levels of depression and stress lower healthy behaviors (19–21).

Prenatal health behaviors are classified as either health-promoting (e.g., exercise, adequate sleep, and nutrition) or health-impairing behaviors (e.g., inappropriate physical activity, smoking, drinking alcohol, and caffeine intake). Health-promoting behaviors necessitate consistent efforts, whereas health-impairing behaviors are reactive to situations and mood; thus, factors influencing health-promoting and health-impairing behaviors may differ (22).

**Figure 1:**
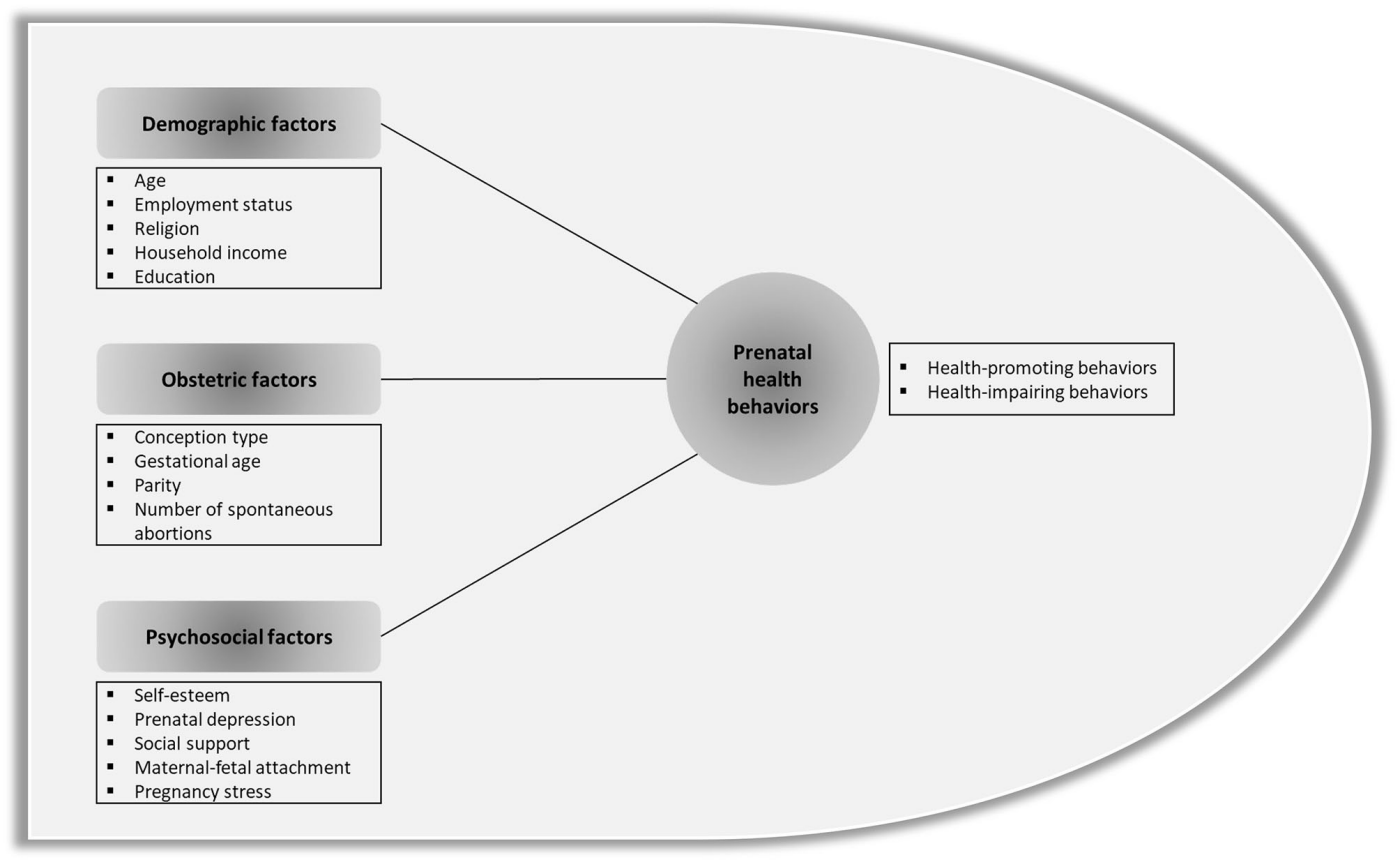
Theoretical framework.

Accordingly, some previous research has identified psychosocial factors influencing both prenatal health-promoting behaviors (23, 24) and prenatal health-impairing behaviors (25). One study even simultaneously reported factors influencing prenatal health-promoting and health-impairing behaviors (26). These previous studies, however, have limitations. For example, they limited the measures of prenatal health-impairing behaviors to smoking and drinking while they limited prenatal health-promoting behaviors to physical activity and exercise. Therefore, the influencing factors should be identified by categorizing health behaviors, including nutrition and eating habits, physical activity, and exposure to hazardous substances, into prenatal health-promoting and health-impairing behaviors. Figure 1 shows the theoretical framework of this study.

This study aimed to determine the association between psychosocial factors and health-promoting and health-impairing behaviors in pregnant women of AMA in Korea. The findings of this study may aid in the development of interventions for prenatal health behavior reinforcement based on the psychosocial factors of pregnant women of AMA in Korea, where AMA rates are high.

## 2. Materials and methods

### 2.1. Design

This was a cross-sectional survey study design.

### 2.2. Sample

Participants in this study were women in low-risk pregnancies aged 35 years or older. The inclusion criteria applied to women who could comprehend and answer the questionnaires in Korean, whereas the exclusion criteria applied to women with multiple pregnancies (e.g., twin and triplet pregnancy) and pregnancy complications [e.g., gestational diabetes mellitus (GDM), gestational hypertensive disorders (GHDs), and placenta previa]. The criteria for calculating the sample size were a level of significance of 0.05, a power of 0.90, and an effect size of 0.15. The sample size was calculated as 202 people using the G^*^Power program 3.1.9.7 (27).

### 2.3. Data collection

Pregnant women of AMA enrolled in response to recruitment notices in online communities (Naver Cafe and Kakao Open Chatting, with the former being the most famous among community-type social network services, and the latter among open chatting platforms in Korea) of pregnant women and at local obstetrics clinics, implying convenience sampling. We gathered data online because the Internet and mobile penetration rates in Korea are very high, so anyone, regardless of education or income level, can easily access the online survey. Pregnant women keen on this study accessed the online survey platform, SurveyMonkey via QR code or Uniform Resource Locator (URL). They reviewed the information (the objective of the study and process of participation, disadvantages/risks and rewards for participation, a statement that the collected data will not be used for any intent other than the purpose of this study, and a statement that participants can quit at any time if they wish to discontinue participation), and then, they agreed to participate in this study. We screened and recruited participants who met the study's selection criteria by inquiring about their age, pregnancy complications, and multiple pregnancies. Additionally, we verified whether the respondent's mobile number to receive the mobile coupon as a reward was duplicated; whether the response time to complete the survey was too short; and whether the estimated day of confinement (EDC) matched gestational weeks. A total of 217 pregnant women volunteered to partake in the study between September and November 2020, but only 207 participants completed the self-report questionnaires via the online survey platform. Our online poll did not include any missing information.

### 2.4. Measures

#### 2.4.1. Prenatal health behaviors

We assessed prenatal health-promoting behaviors and prenatal health-impairing behaviors using the Prenatal Health Behavior Scale (PHBS), including items on sleep, physical activity, nutrition, smoking, and alcohol consumption (28). Studies have validated this scale, originally designed for women in their mid- and late pregnancy, by applying it to women in the initial stages of pregnancy (29). The PHBS assesses health-promoting and health-impairing behavior on a five-point Likert-type scale (from 1 to 5) with 10 items each. The higher score for health-promoting behavior and the lower score for health-impairing behavior denote a higher degree of health behavior. Cronbach's alphas for the original PHBS were 0.75 and 0.59, respectively, for health-promoting and health-impairing behaviors (28). In an earlier study (30) that assessed 20 PHBS items in pregnant women at all pregnancy stages as in this research study, Cronbach's alphas were 0.72 and 0.55 for health-promoting and health-impairing behaviors, respectively. In this study, Cronbach's alphas were 0.76 for health-promoting and 0.70 for health-impairing behaviors.

#### 2.4.2. Demographic and obstetric characteristics

According to research, general, and socioeconomic statuses (e.g., age, education, and income) relate to health behaviors (16, 17). Therefore, we gathered information about participants' age, employment status, religion, household income, and education. We also included the current conception type, gestational age, parity, and the number of spontaneous abortions, all of which link to health behaviors (13, 18, 30). **Table 2** contains information on demographic and obstetric characteristics.

#### 2.4.3. Psychosocial factors

We assessed self-esteem using the Rosenberg Self-esteem Scale (SES) (31). SES consists of 10 items, and a four-point Likert-type scale (from 1 to 4) measures the scores: the higher the score, the higher the self-esteem. Cronbach's alpha for internal consistency reliability of the original version of the SES was 0.85 (31), and it was 0.90, and 0.86 in the Korean version (32) and this study, respectively.

We evaluated prenatal depression using the Edinburgh Postpartum Depression Scale (EPDS) (33). EPDS includes common depressive symptoms related to pregnancies, and it is a viable tool during pregnancy (34); although no scale can examine depression during pregnancy (35), EPDS has been used to measure pregnancy-related depression in numerous kinds of research, including in Korea (36). EPDS comprises 10 items that a four-point Likert-type scale (from 1 to 4) evaluates, where a higher score indicates more severe depression. Cronbach's alpha for EPDS was 0.92 (33), and Cronbach's alphas for the Korean version (37) and this study were 0.85 and 0.82, respectively.

We assessed social support using the Multidimensional Scale of Perceived Social Support (MSPSS) (38) and the Spouse Supportive Behavior Scale (SSBS) (39). These scales were developed a long time ago, but numerous studies have used them as measures of social support until recently. MSPSS includes 12 items, including support from family, friends, and remarkable others. This study restricted family support to family members excluding spouses. As the spouse is intimate with a pregnant woman, we sought to distinguish them from other family members and accurately assess the spouse's support using SSBS. We used 10 items of SSBS, exempting the items of the spouse's supportive behaviors for physical convenience (e.g., “My husband comes home at the right time” and “My husband buys me comfortable clothes and shoes”), to identify the influence of psychosocial factors. We measured these two social support scales on a five-point Likert-type scale (from 1 to 5), with a higher score indicating more social support. Cronbach's alphas for original-version MSPSS (38) and the Korean version (40) were 0.88, and 0.90, respectively, and SSBS was 0.89 (39). This study determined them as 0.92 and 0.91.

We assessed maternal–fetal attachment using the Maternal–Fetal Attachment Scale (MFAS) (41). Although this scale is many years old, recent research still extensively uses the scale. MFAS comprises 24 items. We assessed MFAS on a five-point Likert-type scale (from 1 to 5), with a higher score indicating greater fetal attachment. Cronbach's alphas were 0.85, 0.92, and 0.89 for the original, the Korean version (42), and this study, respectively.

We assessed pregnancy stress using the Korean Pregnancy Stress Scale (PSS) (14). PSS includes 36 items split into seven dimensions: “physical and psychological changes (e.g., ‘I have difficulty breathing'),” “health of the mother and baby (e.g., ‘I am worried about having an abnormal fetus'),” “maternal role (e.g., ‘Becoming a mother is a burden'),” “family support (e.g., ‘I am disappointed that my husband is indifferent to me'),” “healthcare services (e.g., ‘I have difficulty determining prenatal tests'),” “social atmosphere (e.g., ‘If my baby has some problems, our society seems to believe that the mother is the main cause'),” and “coping in daily life (e.g., ‘I eat a balanced diet')”. We used 31 items and six dimensions of them in this study: “physical and psychological changes (eight items),” “health of the mother and baby (six items),” “maternal role (six items),” “family support (four items),” “healthcare services (three items),” “social atmosphere (four items)”. The five excluded items were questionnaires that overlapped with health behavior scales or related to postpartum and childcare; three items of “coping in daily life”; “financial burden about postpartum care”; and “social atmosphere about childcare facility”. PSS was calculated using a five-point Likert-type scale (from 1 to 5), with a higher score indicating greater stress. Cronbach's alphas for the original and this study were 0.85 (14) and 0.90, respectively.

### 2.5. Ethical considerations

Before participant enrollment and data collection, the institutional review board of the author's institution approved this research work (Korea University; No. KUIRB-2020-0244-03). The institutional review board waived the requirement of written informed consent for participation. All participants provided online informed consent, and we obtained the data only from those who voluntarily participated.

### 2.6. Data analysis

We evaluated data using the STATA 16.1 program and measured descriptive statistics for the demographic, obstetric, psychosocial variables, and prenatal health behaviors of samples. The *t*-test and analysis of variance confirmed the differences between pregnant AMA women's prenatal health-promoting and health-impairing behaviors on the basis of demographic, obstetric, and psychological characteristics. We used the Bonferroni method for the *post-hoc* test (43). Pearson's correlation analysis established the relationship between variables. Furthermore, we investigated the association between the factors and prenatal health behaviors using linear regression. We assessed multicollinearity by a variance inflation factor (VIF).

## 3. Results

### 3.1. Demographic, obstetric, and psychosocial characteristics

Table 1 summarizes the demographic, obstetric, and psychosocial characteristics. Most of them have bachelor's degrees. In terms of obstetric variables, more than three-quarters of the subjects had natural conception, and nearly half of them were in their third trimester. More than half of the women were nulliparous, and 62.32% did not have an abortion spontaneously. Among the psychosocial variables, the mean of self-esteem was 3.00 (*SD* 0.40) while the mean of prenatal depression was 1.76 (*SD* 0.42). Maternal–fetal attachment averaged 4.09 (*SD* 0.47). In terms of pregnancy stress, “social atmosphere” was the most significant (mean 3.84), while “family support” was the lowest (mean 1.91). Prenatal health-promoting behaviors averaged 3.50 (global score: 62.5), while health-impairing behaviors averaged 2.18 (global score: 29.5).

**Table 1:** Demographic, obstetric, and psychosocial characteristics of participants (*N* = 207).

**Variables**		***n***	**%**	**M *(SD)***	**Range**
Age (years)	35–39	189	91.30	36.72 (1.94)	35–44
		Above 40	18	8.70		
Employment status	In office	80	38.65		
		Leave	37	17.87		
		Resignation	44	21.26		
		Never employed	46	22.22		
	Religion	Buddhism	93	44.93		
		Christianity	20	9.66		
		Catholics	69	33.33		
		Nothingarian	25	12.08		
Monthly household income ($^*^)	Below 2,000	9	4.35		
		2,000~4,000	70	33.82		
		4,000~6,000	62	29.95		
		6,000~8,000	35	16.91		
		Above 8,000	31	14.97		
Education	High school	8	3.87		
		College (Associate degree)	27	13.04		
		University (Bachelor's degree)	123	59.42		
		Graduate (Master's and Doctoral degree)	49	23.67		
	Conception type	Natural	160	77.29		
			Artificial	47	22.71		
	Gestational age (days)	1st trimester (-97)	36	17.40	173.85 (70.22)	32–275
			2nd trimester (98–195)	76	36.71		
			3rd trimester (196-)	95	45.89		
Parity		Nulliparous (0)	142	68.60	0.39 (0.63)	0–3
		Multiparous (≥1)	65	31.40		
Spontaneous abortions	0	129	62.32	0.55 (0.85)	0–5
		1–2	70	33.82		
	≥3	8	3.86		
Self-esteem				3.00 (0.40)	1.6–4
Prenatal depression				1.76 (0.42)	1–2.8
Social support	Support of spouse			4.10 (0.62)	1.3–5
		Support of family			4.22 (0.74)	1–5
	Support of friends			3.74 (0.82)	1–5
	Support of significant others			3.36 (1.14)	1–5
Maternal-fetal attachment				4.09 (0.47)	2.7–5
Pregnancy stress				3.02 (0.57)	1.7–4.5
	PSS 1			3.56 (0.70)	1.5–5
	PSS 2			3.13 (1.07)	1–5
	PSS 3			2.68 (0.94)	1–5
	PSS 4			1.91 (0.77)	1–4.8
	PSS 5			2.37 (0.94)	1–5
	PSS 6			3.84 (0.69)	2–5
Prenatal health-promoting behavior				3.50 (0.58)	1.3–4.8
Prenatal health-impairing behavior				2.18 (0.48)	1–4.4

^*^1$ = 1,000 Won (Korean), PSS 1, physical and psychological changes; PSS 2, health of the mother and baby; PSS 3, maternal role; PSS 4, family support; PSS 5, healthcare services; PSS 6, social atmosphere.

### 3.2. Prenatal health behavior according to demographic, obstetric, and psychosocial characteristics

Table 2 shows the prenatal health behavior according to demographic, obstetric, and psychosocial characteristics. In demographic and obstetric variables, education (*F* = 4.57, *p* = 0.004), parity (*t* = 6.92, *p* = 0.009), and the number of spontaneous abortions (*t* = 3.40, *p* = 0.035) were significantly different in prenatal health-promoting behaviors. Meanwhile, conception type (*t* = 12.96, *p* < 0.001) and parity (*t* = 13.61, *p* < 0.001) differed significantly in prenatal health-impairing behaviors. A *post-hoc* analysis was performed using the Bonferroni method. Statistically different demographic and obstetric variables were used to adjust the regression model.

**Table 2:** Prenatal health behavior according to demographic, obstetric, and psychosocial variables (*N* = 207).

**Variables**		**Prenatal health-promoting behavior**				**Prenatal health-impairing behavior**			
		**M** ±**SD**	**t/F**	**r**	***p***	**M** ±**SD**	**t/F**	**r**	***p***
Age				0.00	0.956			−0.13	0.062
Employment status	In-office	3.4 ± 0.6	0.22		0.886	2.2 ± 0.5	0.22		0.886
		Leave		3.5 ± 0.6							2.2 ± 0.6			
		Resignation		3.5 ± 0.6							2.2 ± 0.5			
		Never employed		3.6 ± 0.5							2.1 ± 0.4			
Religion	Buddhism	3.4 ± 0.6		1.55				0.203		2.2 ± 0.5	1.55		0.203
		Christianity	3.4 ± 0.6						2.3 ± 0.6					
		Catholics	3.6 ± 0.5						2.1 ± 0.5					
		Nothingarian	3.6 ± 0.5						2.2 ± 0.4					
Monthly household income ($^*^)	Below 2,000	3.8 ± 0.6	1.17		0.326	1.9 ± 0.5	1.17		0.326
		2,000~4,000		3.5 ± 0.7							2.2 ± 0.5			
		4,000~6,000		3.4 ± 0.6							2.2 ± 0.4			
		6,000~8,000		3.5 ± 0.5							2.1 ± 0.4			
	Above 8,000	3.6 ± 0.4				2.2 ± 0.4			
Education^†^	High school^a^	3.2 ± 0.5	4.57b <dc <d		0.004^**^	2.0 ± 0.3	1.52		0.211
		College^b^		3.3 ± 0.6							2.2 ± 0.5			
		University^c^		3.5 ± 0.6							2.2 ± 0.5			
		Graduate^d^		3.7 ± 0.5							2.1 ± 0.5			
Current conception type	Natural	3.5 ± 0.6	0.35		0.552	2.2 ± 0.5	12.96		<0.001^**^
		Artificial		3.5 ± 0.7							2.0 ± 0.5			
Gestational age	1st trimester	3.5 ± 0.7	2.43		0.091	2.1 ± 0.5	1.31		0.271
		2nd trimester		3.6 ± 0.5							2.2 ± 0.5			
		3rd trimester		3.4 ± 0.6							2.2 ± 0.5			
Parity	Nulliparous	3.6 ± 0.6	6.92		0.009^**^	2.1 ± 0.4	13.61		<0.001^**^
		Multiparous		3.3 ± 0.6							2.4 ± 0.5			
Number of spontaneous abortion^†^	0^a^	3.6 ± 0.6	3.40b <a		0.035^*^	2.1 ± 0.4	1.32		0.269
		1–2^b^		3.4 ± 0.5							2.3 ± 0.5			
		3+^c^		3.5 ± 0.6							2.2 ± 0.5			
Self-esteem				0.28	<0.001^**^			−0.21	0.002^**^
Prenatal depression				−0.20	0.003^**^			0.27	<0.001^**^
Social support	Support of spouse			0.18	0.009^**^			−0.24	<0.001^**^
		Support of family			0.14	0.047^*^			−0.04	0.550
		Support of friends			0.17	0.016^*^			−0.04	0.580
		Support of significant others			0.16	0.022^*^			0.00	0.996
	Maternal-fetal attachment			0.44	<0.001^**^			−0.20	0.004^**^
Pregnancy stress	PSS 1			−0.13	0.157			0.26	<0.001^**^
	PSS 2			−0.10	0.157			0.24	<0.001^**^
	PSS 3			−0.17	0.048^*^			0.36	<0.001^**^
	PSS 4			−0.15	0.028^*^			0.34	<0.001^**^
	PSS 5			−0.07	0.291			0.20	0.003^**^
	PSS 6			0.16	0.025^*^			0.06	0.407

1$ = 1,000 Won (Korean), PSS 1, physical and psychological changes; PSS 2, health of the mother and baby; PSS 3, maternal role; PSS 4, family support; PSS 5, healthcare services; PSS 6, social atmosphere; ^*^p < 0.05, ^**^ *p* < 0.01. ^†^The a, b, c, and d are indicated for the *post-hoc* test results. The following right cell included the *post-hoc* test results.

In psychosocial variables, prenatal health-promoting behaviors significantly positively linked to self-esteem (r = 0.28, *p* = 0.001), prenatal depression (r = 0.20, *p* = 0.003), support of spouse (r = 0.18, *p* = 0.009), support of family (r = 0.14, *p* = 0.047), support of friends (r = 0.17, *p* = 0.016), support of significant others (r = 0.16, *p* = 0.022), and maternal–fetal attachment (r = 0.44). Furthermore, prenatal depression (r =-0.20, *p* = 0.003) significantly negatively linked to prenatal health-promoting behaviors. Among pregnancy stressors, “maternal role” (r = −0.17, *p* = 0.048) and “family support” (r = −0.15, *p* = 0.028) significantly negatively linked to prenatal health-promoting behaviors. Furthermore, prenatal health-promoting behaviors positively linked to the social environment (r = 0.16, *p* = 0.025).

Prenatal health-impairing behaviors significantly positively associated with prenatal depression (*r* = 0.27, *p* < 0.001). However, there was a positive relationship between prenatal health-impairing behaviors and self-esteem (*r* = −0.21, *p* = 0.002), spouse support (*r* = −0.24, *p* < 0.001), and maternal–fetal attachment (*r* = −0.20, *p* = 0.004). Furthermore, in pregnancy stress, “physical and psychological change” (*r* = 0.26, *p* < 0.001), “health of the mother and baby” (*r* = 0.24, *p* < 0.001), “maternal role” (*r* = 0.36, *p* < 0.001), “family support” (*r* = 0.34, *p* < 0.001), and “healthcare services” (*r* = 0.20, *p* = 0.003) significantly positively associated with prenatal health-impairing behaviors.

### 3.3. Factors associated with prenatal health behaviors

Table 3 shows the factors significantly associated with prenatal health-promoting behaviors. We adjusted demographic and obstetric variables, including education, parity, and spontaneous abortions. Psychosocial factors explained prenatal health-promoting behaviors, adjusted *R*^2^ = 0.24, *F* (degree of freedom) = 5.09, *p* < 0.001. Maternal–fetal attachment (β = 0.43, *p* < 0.001) and “social atmosphere” of pregnancy stress (β = 0.13, *p* = 0.047) significantly associated with prenatal health-promoting behaviors. The variance inflation factors (VIFs) ranged from 1.17 to 7.18.

**Table 3:** Linear regression analysis summary for psychosocial factors associated with prenatal health-promoting behavior (*N* = 207).

**Variables**		**β Estimate**	**SE**	***p***
Self-esteem		0.17	0.13	0.057
Prenatal depression		−0.06	0.12	0.521
Social support	Support of spouse	−0.04	0.10	0.723
		Support of family	−0.04	0.06	0.616
		Support of friends	−0.06	0.06	0.483
Support of significant others		0.00	0.04	0.965
Maternal-fetal attachment		0.43	0.09	<0.001^**^
Pregnancy stress	PSS 3	0.08	0.05	0.375
		PSS 4	0.04	0.08	0.719
		PSS 6	0.13	0.06	0.047^*^

Adjusted for education, parity, and spontaneous abortions. PSS 3, maternal role; PSS 4, family support; PSS 6, social atmosphere. R^2^ = 0.30, adj R^2^ = 0.24; ^*^p < 0.05, ^**^p < 0.01.

Table 4 shows the factors associated with prenatal health-impairing behaviors. We adjusted obstetric variables, including current conception type and parity. Psychosocial factors explained prenatal health-impairing behaviors, adjusted *R*^2^ = 0.22, *F* (degree of freedom) = 6.28, *p* < 0.001. Artificial conception (β = −0.16, *p* = 0.011), multipara (β = 0.23, *p* = 0.001), and “maternal role” of pregnancy stress (β = 0.27, *p* = 0.003) significantly associated with prenatal health-impairing behaviors. The VIF was 1.08–3.09.

**Table 4:** Linear regression analysis summary for psychosocial factors associated with prenatal health-impairing behavior (*N* = 207).

**Variables**		**β Estimate**	**SE**	***p***
Self-esteem		0.02	0.10	0.809
Prenatal depression		0.01	0.11	0.937
Social support	Support of spouse	0.04	0.08	0.712
Maternal-fetal attachment		0.02	0.08	0.757
Pregnancy stress	PSS 1	0.01	0.05	0.936
		PSS 2	0.12	0.03	0.117
		PSS 3	0.27	0.05	0.003^**^
		PSS 4	0.15	0.07	0.159
	PSS 5	0.00	0.04	0.952

Adjusted for current conception type and parity. PSS 1, physical and psychological changes; PSS 2, health of the mother and baby; PSS 3, maternal role; PSS 4, family support; PSS 5, healthcare services. R^2^ = 0.26, adj R^2^ = 0.22; ^**^p < 0.01.

## 4. Discussion

Given that Korea has the highest mean age of women at childbirth among the OECD countries, this study was conducted on Korean pregnant women of AMA. This study is also one of the first to examine the influence of demographic, obstetric, and psychosocial factors on health behaviors in pregnant women of AMA by distinguishing health-promoting behaviors and health-impairing behaviors. As a result, the significant variables that were associated with health-promoting behaviors were maternal–fetal attachment and “social atmosphere” of pregnancy stress, whereas conception type, parity, and “maternal role” of pregnancy stress were significantly associated with health-impairing behaviors.

The global score means of prenatal health-promoting and health-impairing behaviors in this study were 62.5 and 29.5, respectively, and in the study using the same tool, they were 62.3 and 25.5, respectively (30). This study demonstrated slightly higher health-promoting behaviors than Pope et al. (30) did, thought to be because older pregnant women engaged in healthier behaviors than younger women did. The mean age of the sample of this study and that of the previous one was 36.72 years and 32.38 years, respectively. Meanwhile, we found health-impairing behaviors to be more prevalent but found behaviors that seriously harm maternal and fetal health, such as drinking and smoking, to be less prevalent. It may influence the cultural diversity of the health behaviors of pregnant women. In addition, cultural differences may affect the internal consistency of health behaviors. Yet, Cronbach's alpha is higher in this study than it is in others. In an earlier study with the same measurement but different item numbers (29), Cronbach's alphas for health-promoting and health-impairing behaviors were 0.93 and 0.93, respectively.

There were no demographic factors that significantly influenced prenatal health-promoting and health-impairing behaviors. However, education levels demonstrated a significant difference in prenatal health-promoting behaviors. In this study, a majority of the sample had high education levels (college, 13.0%; university, 59.4%; and graduate, 23.7%). Researchers assume Korean young adults have the highest level of education among OECD countries (44), and highly educated women frequently postpone marriage and pregnancy in favor of their careers. Generally, we believe that the more educated the people, the healthier their behaviors. Furthermore, pregnant women with a high level of education may engage in more healthy behaviors because they have a higher socioeconomic status and are more knowledgeable about prenatal health than those with a lower level of education would be.

This study found no significant obstetric factors influencing prenatal health-promoting behaviors, but current conception type and parity had a significant impact on prenatal health-impairing behaviors. First, women who had artificial conception are less likely to engage in health-impairing behaviors than women who had natural conception. Women who became pregnant using assisted reproductive technology experienced fear and anxiety about possible fetal loss (45). Thus, it is a belief that pregnant women with artificial conception avoid health-impairing behaviors for the safety of their fetus and the maintenance of pregnancy. Furthermore, pregnant AMA women may engage in more protective behaviors to maintain pregnancy than younger women might, given that reproductive functions and fertility decline with age. According to the findings, pregnant women with artificial conception require encouragement and support to avoid engaging in risky behaviors, as well as relief from anxiety about miscarriage and fetal health. Second, multiparous pregnant women engaged in more unhealthy behaviors than nulliparous women did. Nulliparous pregnant women are generally more anxious than multiparous pregnant women (46). Furthermore, primigravidas at AMA are more likely to engage in health-promoting behaviors than younger primigravidas (47). Thus, multiparous women may be less conscious of the risk of health-impairing behaviors during pregnancy because they have already undergone pregnancy and childbirth, and possibly, they gave birth at a younger age. Pregnant women with childbirth experience need emphasizing that they should avoid compromising their health even if they have previously experienced pregnancy and childbirth because the risk of pregnancy complications increases with maternal age.

Pregnant women with high fetal attachment exhibited more health-promoting behaviors than those with low fetal attachment according to this study's analysis of psychosocial factors. Likewise, a meta-analysis reported that maternal–fetal attachment is a strong predictor of prenatal health behavior (15). However, earlier research (48, 49) revealed that prenatal attachment influences health-impairing behaviors, such as smoking, in contrast to the findings of this study, which did not demonstrate a significant effect of fetal attachment on health-impairing behaviors. However, it was only “giving of self,” a subscale of fetal attachment, and not the total fetal attachment score. It might be because pregnant women who have a high fetal attachment rate tend to engage in more vigorous prenatal health-promoting behaviors than prenatal health-impairing ones. Fetal attachment has a positive effect on health behaviors and ultimately, fetal health (50); thus, nurses can provide much-needed interventions to enhance maternal–fetal attachment by recognizing the fetus as an entity and strengthening the relationships and interactions with the fetus in pregnant women of AMA.

Stress during pregnancy was another important component as it had an impact on both prenatal health-promoting behaviors and health-impairing behaviors. “Social atmosphere,” the social context of motherhood and childcare, induced more stress, which was related to a greater practice of health-promoting behaviors. On the other hand, the maternal role involved more stress, associated with a higher practice of health-impairing behaviors. These contradictory findings, which depend on the factors causing pregnancy stress, are brought on by the pervasive social belief that women in Korean society are in charge of raising children (51). Women are under social pressure that, as mothers, they must devote their time and effort to childcare; they are also afraid of social judgments comparing themselves with other mothers (52). In particular, Koreans are extremely sensitive to the opinions of people around them and their social views of them (53). As a result, peer pressure may motivate expectant mothers to maintain their healthy behaviors. In contrast, this societal pressure can lead to undue stress by parental duties and make expectant mothers feel powerless and burdened, which can result in behaviors that harm their health. Therefore, pregnant women of AMA need interventions for lowering pregnancy stress related to the mother's role.

### 4.1. Implications

On the basis of these results, we propose three implication points for practice and policy. First, it is necessary to assess the status of stress during pregnancy. In pregnant women of AMA, pregnancy stress affects both health-promoting and health-impairing behaviors. In Korea, public health centers screen for prenatal depression and link it to intervention programs because of the emphasis on the significance of mental health; however, they do not assess for prenatal stress. Thus, it is important to assess pregnancy stress, one aspect of mental health, and to employ stress reduction strategies as appropriate. Second, we should consider cultural differences when developing prenatal health promotion programs. Contrary to earlier research that claimed that more stress led to poorer health behavior, pregnant women of AMA engaged in more health-promoting activities because of higher pregnancy stress related to the social atmosphere. The outcome could be a reflection of Korean culture and social norms, which strongly encourage pregnant women to adhere to prenatal health behavior recommendations. Thus, it is important to take cultural variations into account rather than universally applying the findings to all nations. Third, in the contents of the health behavior promoting program for pregnant women of AMA, it is suggested to consider not only emphasizing the necessity and method of prenatal health behaviors but also enhancing fetal attachment by providing an opportunity to interact with the fetus. Fourth, childbirth experiences, among adjusted obstetric variables, warrant consideration in developing health promotion programs for pregnant women. In this study, multiparous women tend to do prenatal health-impairing behaviors more than nulliparous women do. As a result, multiparous pregnant women require monitoring for unhealthy habits, and the program should highlight the significance of health-promoting activities.

### 4.2. Limitations

This study has some limitations. First, this study has a cross-sectional study design; thus, identifying the causal relationships between variables through data analysis was difficult. Additionally, it was unable to obtain data on pre-pregnancy health practices. Second, this study needs consideration when generalizing and applying the data to a country. Researchers must consider each country's cultural traits while implementing our study's findings. Third, we used an online survey to alleviate the drawbacks of convenience sampling; nonetheless, as Internet surveys are unable to contain representative samples, a type 1 error may have occurred. Future research will need to conduct a nationwide survey with systematic sampling in order to obtain an accurate population representation. Finally, we were unable to account for all confounding variables, such as pre-pregnancy health behaviors, including, smoking, alcohol consumption, exercise, and diet.

## 5. Conclusion

This study extracted the factors affecting the health behaviors of pregnant women of AMA. Maternal–fetal attachment, and stress induced by social atmospheres, such as expectations for motherhood and childcare, influenced prenatal health-promoting behaviors. Whereas the current conception type, parity, and maternal role stress influenced prenatal health-impairing behaviors. We discussed the implications in light of these findings and considered childbirth experience and cultural variations while creating health promotion initiatives for expectant mothers. Additionally, it is important to monitor pregnancy stress levels. These recommendations can help create plans for healthy behavior in pregnant women of AMA.

## Data availability statement

The original contributions presented in the study are included in the article/supplementary material, further inquiries can be directed to the corresponding author.

## Ethics statement

The studies involving human participants were reviewed and approved by Korea University Institutional Review Board. The Ethics Committee waived the requirement of written informed consent for participation.

## Author contributions

SJ conceptualized and designed the study, collected data, contributed to analyses, and wrote the manuscript. WN conceptualized the study, analyzed the data, critically discussed the results, and wrote the manuscript. All authors contributed to the manuscript revision and reading and approved the submitted version.
